# Detection of Intracellular Gold Nanoparticles: An Overview

**DOI:** 10.3390/ma11060882

**Published:** 2018-05-24

**Authors:** Mario D’Acunto

**Affiliations:** Consiglio Nazionale delle Ricerche, Istituto di Biofisica, CNR-IBF, via Moruzzi 1, 56124 Pisa, Italy; mario.dacunto@pi.ibf.cnr.it

**Keywords:** gold nanoparticles, cellular uptake mechanism, Dark Field Microscopy, Surface-enhanced Raman spectroscopy, Scanning Near-Optical Microscopy

## Abstract

Photothermal therapy (PTT) takes advantage of unique properties of gold nanoparticles (AuNPs) (nanospheres, nanoshells (AuNSs), nanorods (AuNRs)) to destroy cancer cells or tumor tissues. This is made possible thanks principally to both to the so-called near-infrared biological transparency window, characterized by wavelengths falling in the range 700–1100 nm, where light has its maximum depth of penetration in tissue, and to the efficiency of cellular uptake mechanisms of AuNPs. Consequently, the possible identification of intracellular AuNPs plays a key role for estimating the effectiveness of PTT treatments. Here, we review the recognized detection techniques of such intracellular probes with a special emphasis to the exploitation of near-infrared biological transparency window.

## 1. Introduction

Photothermal therapy (PTT) is a non-invasive treatment for the therapy for many diseases, in a special manner, for cancer treatment [1]. PTT is also one of the most promising technologies to arrest the expansion of cancerous cells with reduced toxicity due to its effective ability to destroy the cancer cells locally [2]. It is based on the thermal therapy induced by a laser illumination able to activate the heat production by internalized nanoparticles (NPs) in cancer cells [3,4,5,6,7,8,9,10]. The accumulation of near-infrared absorbing NPs in tumor sites enormously increases the efficiency of PTT through effective conversion of light into heat energy [11].

A PTT treatment is based on the effect increasing temperatures have on living cells, and it is commonly accepted that above 42 °C cell viability is strongly reduced. In fact, hyperthermia effects can range from moderate denaturation of blood and extracellular proteins to induction of apoptosis and, above 50 °C, to cell death and tissue ablation [12]. PTT has been widely used via either direct irradiation or suitable temperature vectors, such as metal NPs [13]. In NP-mediated PTT cancer treatments, NPs heat up cancerous cells beyond their temperature tolerance limits, which are lower than normal healthy tissue due to their poor blood supply, killing them selectively. This can be achieved by exposing the entire patient or the targeted area to an alternating current magnetic field, an intense light source or radiofrequencies, which will cause the NPs to heat up and induce thermal ablation of the tumor. One of the most widespread examples of hyperthermia mediated by NPs, magnetic NPs have been introduced in the body through magnetic delivery systems or local injection to the affected area [14]. The first in vivo Phase II clinical trials of magnetic NP hyperthermia were undertaken in Germany in 2005 [15] by injecting the prostate of cancer patients with biocompatible magnetite NPs. Successful results were obtained using minimally invasive ablation of the tumor in an AC magnetic field after several sessions.

Endocytosis is, hence, a fundamental biological process used by cells to internalize NPs, where, normally, NP size can affect the uptake efficiency, intracellular kinetics and the overall internalization mechanism [16,17,18]. A size-dependent uptake in different cell lines has been observed for AuNP [19], as well as iron oxide NPs [20]. Consequently, the understanding of the endocytosis process and the efficiency of cellular uptake is based on the ability to identify internalized NPs inside cells.

Several instrumentations are commonly used to carry out this task. One of the most widely employed method of detecting AuNPs is through fluorescence with the use of fluorescent molecules attached to the gold surface or by fluorescent gold core [21,22,23]. The fluorescence can be estimated by wild field microscopy [24], or, for enhanced resolution, by confocal laser scanning microscopy [25]. Transmission electron microscopy (TEM) is a widely used microscopy technique for the intracellular detection of metal NPs bigger than 5 nm [26,27]. Although TEM is a powerful technique that allows the imaging of metal NPs in cellular components or organelles, nevertheless, it requires time-consuming sample processing. Differential interference contrast (DIC) microscopy is an optical microscopy technique working on the principle of interferometry to gain information about the optical path length analogously to phase contract microscopy but without the bright diffraction halo. DIC has been utilized to study the endocytosis of functionalized AuNPs with diameters falling in the range 20–80 nm [28,29,30].

Surface-enhanced Raman spectroscopy (SERS) is another popular method for live cell investigations [31]. SERS is label-free, highly sensitive and non-destructive method which allows for molecular identification. In fact, a Raman spectral image is produced by various molecular vibrations and offers a vibrational fingerprint of a molecule. The Raman scattering signal can be greatly increased for the molecules, which are spatially close to metal nanostructures. AuNPs are able to enhance the performances of internalized molecules by 10^10^ to 10^15^ folds, allowing spectroscopic detection of single molecules [32]. Due to its importance in the detection of intracellular AuNPs, SERS methods and applications will be more detailed in Section 4.

Other than SERS, different other techniques based on the plasmonic properties have been developed in the last decade. Photothermal microscopy is a technique exploiting two overlapping laser beams, one that triggers localized small variations of temperature (approximately 1–2 °C) in presence of nano-absorbers, and the other detects possible changes in temperature [33]. In living cells, this technique is able to detect 5 nm AuNPs [34] and 10 nm × 6 nm AuNRs [35]. A recent version has been developed for video-rate tracking imaging of single NP photothermal [36]. Analogously to photothermal imaging, photoacoustic microscopy exploits the conversion of absorbed light into heat producing an ultrasonic wave via thermoelastic expansion. High-resolution dynamic contrast-enhanced photoacoustic imaging in vivo has been recently demonstrated using acoustic nanomodulators [37]. In turn, Scanning Near-Optical Microscopy (SNOM) has been employed to detect and localize 120–150 nm AuNPs (nanoshells) absorbing light in the near-infrared range exploiting the biological transparency window [38,39,40,41]. By default, in the rest of the text we will indicate the NPs of spherical geometry with the term AuNP, and we will specify the other NP geometries (AuNS or AuNR) when necessary.

The review is organized as follows. We describe the endocytosis and cellular uptake mechanisms of bared or functionalized AuNPs in Section 2. In Section 3, we analyze the optical and photothermal properties of AuNPs describing the plasmonic properties of such NPs. Finally, in Section 4 we focus the attention on some experimental techniques for detection and tracking of AuNPs, principally on Dark Field Microscopy (DFM), SERS, and SNOM. In turn, the theranostics employment of non-metal materials in PTT is briefly described. 

## 2. Endocytosis and Cellular Uptake Mechanisms of AuNPs

All types of cells in the body exploit the endocytosis or exocytosis processes to communicate with the biological environments [42]. Such processes are energy-based processes though which cells internalize (or externalize) ions, nutrients and signaling molecules to interact with other cells [43]. In particular, focusing the attention on the only endocytosis process, the cells obtain energy through specific pathways, typically classified into clathrin- and caveolae-mediated endocytosis, phagocytosis (internalization of large particles ~500 nm), and non-specific pathways such as micropinocytosis (internalization of fluids and particles together and large vesicles ~200–500 nm), and pinocytosis (absorption of extracellular fluids, small molecules, and small vesicles <200 nm) [44].

When NPs are administered for biomedical application or absorbed via airways due to pollution into the body, in this case, the endocytosis of such NPs depend by some parameters, such as size, shape and surface chemistry, and by cell type [16]. Therefore, the study of endocytosis of NPs meets great interest in nanomedicine, and, for similar reasons, in nanotoxicology [45]. In this review, we consider only AuNPs systematically administered for PTT. Various types of cells through the plasma membrane internalize AuNPs circulating in the bloodstream. The plasma membrane is a selectively permeable membrane that allow the passage of objects the size of hundreds of nanometers thanks to the fact that on the membrane can open and close pores without destroying the membrane itself. The growth of pores on the cell membrane is controlled by the rate at which the membrane elastic energy is dissipated [46]. Since the viscosity of the membrane is five orders of magnitude larger than the water surrounding the membrane, most of the energy dissipation is confined to the membrane interior [47]. The energetically stability of such membrane pores discriminate the particle to be internalized in terms of size and shape of the AuNPs (as well as all the other types of NPs) [48,49,50]. AuNPs with varying core size are prepared by the reduction of gold salts in the presence of stabilizing agents both to control growth and to avoid nanoparticle agglomeration.

In addition, another basic property of internalized AuNPs, other than size and shape, is their surface chemistry [16]. Since biological systems consist of numerous biomolecules with various charges, surface chemistry include the chemical composition and the surface charge of the NPs. AuNPs can be easily functionalized by anchoring thiol linkers in the gold surface. A wide variety of functionalized bionanoconjugates includes peptides, proteins, antibodies, oligosaccharides, nucleic acids, etc. [51,52]. AuNPs so functionalized act as a multifunctional platform for both diagnostic and therapeutic purposes, commonly recognized as theranostics [53,54,55]. This is because functionalized AuNPs exploit the optical properties of gold and the biochemistry of the functionalizing coating. In the next section, we detail the optical and correspondent photothermal properties of the various types of AuNPs.

## 3. Optical and Photothermal Properties of AuNPs

**Figure 1:**
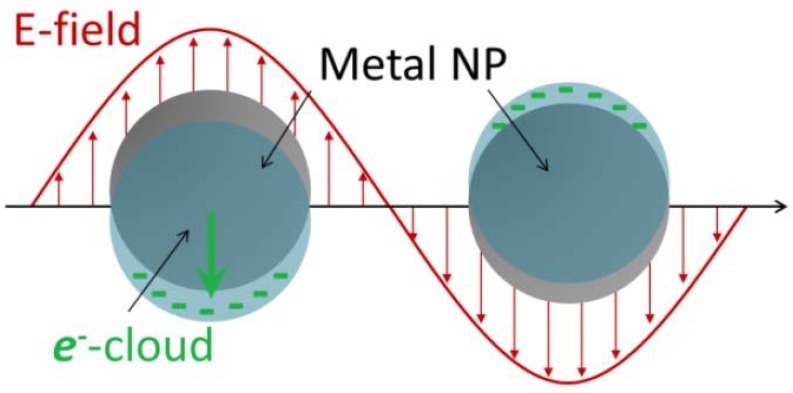
Schematic example of plasmon oscillation for a spherical gold NP showing the displacement of the conduction electron charged cloud relative to the core under the incoming oscillating electric field.

Optical and photothermal properties are strictly connected one each other. This connection plays a basic role not only for the thermal therapies, but also for the intracellular detection and tracking, being all the intracellular detection techniques of metallic NPs based on optical and subsequent thermal behavior. The optical properties of the AuNPs, analogously to other metallic NPs, can be described via the localized surface plasmon resonances (LSPRs). Once irradiated at the LSPR, the electromagnetic energy is absorbed and dissipated as heat into the surrounding media via diffusive process. The temperature of irradiated metallic NPs can easily reach hundreds of degrees Celsius enabling application not only for photothermal therapies, but also for chemical catalysis, drug delivery and vapor generation [56,57,58]. An LSPR can be described as a coherent oscillation of the free carrier gas in a metallic NP. The free carriers are confined to a small volume localizing the surface plasmon to the single NP. Hence, the incoming electromagnetic (e.m.) wave drives the free carriers to oscillations, following the frequency of the incoming electric field of the light wave, Figure 1, at the NP surface, with the positive nuclei acting as a restoring force. In the bulk, a classical harmonic oscillator model well describes the behavior of the free carriers.

In the general case, the eigenfrequency of a LSPR is determined by many factors, including the concentration and the effective mass of conduction electrons, the shape, structure, and size of particles, interaction between particles, and the influence of environment.

However, for an elementary description of NP optics it is sufficient to use a combination of the usual dipole Mie theory and the Drude theory [4,38,59,60]. Adopting the Mie-Drude picture, the absorption and scattering of light by a small particle are determined by its electrostatic polarizability *α*, which can be calculated by using the optical dielectric function *ε*(*ω*). As the particle radius *R* is decreased to the value comparable with the electron mean free path, deviations of the phenomenological dielectric function of the particle from the bulk values can be expected. For a small particle with a radius *R* = (3*V*/4*π*)^1/3^, where *V* is the particle volume, embedded in a homogeneous dielectric medium with the permittivity *ε_m_*, we have the following expressions for the extinction, absorption and scattering cross sections [59], respectively:

(1) Qabs=kπR2Im(α)=4kRIm(ε(ω)−εmε(ω)+2εm) and Qsca=k46π2R2|α|2=8πk4R4|ε(ω)−εmε(ω)+2εm|2

where the expression for the polarizability has been explicated. In the case of NSs, the polarizability changes as [61,62]

(2) $$\alpha =4\pi {\left(r+d\right)}^{3}\frac{\left(\varepsilon_{s}\left(\omega \right)-\varepsilon_{m}\right)\left(\varepsilon_{c}+2\varepsilon_{s}\left(\omega \right)\right)+{\left(\frac{r}{r+d}\right)}^{3}\left(\varepsilon_{c}-\varepsilon_{s}\left(\omega \right)\right)\left(\varepsilon_{m}+2\varepsilon_{s}\left(\omega \right)\right)}{\left(\varepsilon_{s}\left(\omega \right)+2\varepsilon_{m}\right)\left(\varepsilon_{c}+2\varepsilon_{s}\left(\omega \right)\right)+{\left(\frac{r}{r+d}\right)}^{3}\left(\varepsilon_{c}-\varepsilon_{s}\left(\omega \right)\right)\left(2\varepsilon_{s}\left(\omega \right)-2\varepsilon_{m}\right)}$$

where *r* is the radius of the core, *d* is dimension of the gold layer, so that the NS radius is *R* = *r* + *d* and Equation (1) must be changed accordingly.

It has been demonstrated that AuNSs with gold layers less than 40 nm, the absorption efficiency is more higher than scattering efficiency, *Q_ab_*_s_ > *Q_sca_*, and the absorption efficiency is greater the scattering efficiency, the smaller the thickness of the shell layer [40]. From the point of view of the applicability for thermal therapy, this characteristic large absorption cross section make AuNS excellent candidate as heat sources. The temperature generated by an AuNS irradiate with a plasmon resonant wavelength can be expressed by the relation (originally this expression was introduced for gold nanostars) [63]

(3) $$T≅I\frac{Q_{abs}R}{\kappa }$$

where *I* is the irradiance of the incoming beam, *R* the AuNS radius, and *κ* is the thermal conductivity of the AuNS, or alternatively the surrounding medium.

Heat transfer between a NP and its cellular environment is carried out predominantly by radiation, the cellular environment being assumed to be connected to a large environment equivalently to a thermal bath of temperature *T_cell_*. When the AuNP is illuminated in steady-state by an external laser beam, the power absorbed (assuming also energy conservation) by the AuNP equals the difference between the power *P_rad_*(*T*) thermally emitted by the AuNP at temperature *T* and the power emitted by the AuNP at the bath temperature *T_cell_*

(4) $$P_{abs}=P_{rad}\left(T\right)-P_{rad}\left(T_{cell}\right)$$

The power absorbed by the AuNP is nearly proportional to the incident power carried by the external beam by the relation [64]

(5) $$P_{abs}=\pi R^{2}Q_{abs}\left(\lambda ,T\right)I_{inc}$$

where *Q_abs_* is the absorption efficiency given by Equation (1), *I_inc_* is the intensity of the external light beam, and the prefactor denotes the geometrical cross-section of the AuNP considered spherical in this case. The power thermally emitted by the AuNP at temperature *T* is given by the relation [65]

(6) $$P_{rad}\left(T\right)=8\pi R^{2}\int \frac{\hbar c^{2}e_{\lambda_{e}}}{\left[\exp\left(2\pi \hbar c/\lambda_{e}k_{B}T\right)-1\right]}\frac{d\lambda_{e}}{\lambda_{e}^{5}}$$

where ℏ is the Planck’s constant, *c* is the speed of light in vacuum, *e_λ_* is the thermal emissivity and *k_B_* is the Boltzmann’s constant. It is reasonable to assume that the thermal emissivity is equal to absorption efficiency *Q_abs_* of the AuNP, hence inserting Equations (5) and (6) in Equation (4), we obtain

(7) $$Q_{abs}\left(\lambda ,T\right)I_{inc}=8\pi \int \frac{\hbar c^{2}Q_{abs}\left(\lambda_{e},T\right)}{\exp\left(2\pi \hbar c/\lambda_{e}k_{B}T\right)}\frac{d\lambda_{e}}{\lambda_{e}^{5}}-I_{rad}\left(T_{cell}\right)$$

where $I_{rad}\left(T_{cell}\right)=P_{rad}\left(T_{cell}\right)/\pi r^{2}$ is the power density emitted by the AuNP before the illumination at the bath temperature *T_cell_*, and the denominator denotes the geometrical factor for a spherical NP, for NRs or NSs this factor should be changed accordingly. The temperature *T* generated by the AuNP can be obtained numerically by solving Equation (7) using an incremental-iterative procedure with adaptive step-size *dT* and initial value coincident to *T_cell_*. To this purpose we need a known power density *I_inc_*, an incident illumination with fixed wavelength *λ*, and a fixed angle of incidence. To destroy a tumor cell for uptaked AuNP is enough to reach 44–45 °C. The exploitation of infrared transparency window, 750–1100 nm, requires the utilization of AuNSs being such type of NPs for dimensions and core-shell composition able to absorb light in such wavelength range [40,66,67,68].

Due to the infrared transparency window region, the heating of a cell, where previously an AuNS has been internalized and illuminated by a laser with a near-infrared wavelength, can be quantified assuming that only the AuNS generates heat. In the environment composed by the cell surrounding the AuNS, the heat conduction is described by [69]

(8) $$\rho C_{p}\frac{\partial T\left(r,t\right)}{\partial t}=\nabla k\left(r\right)\nabla T\left(r,t\right)+Q\left(r,t\right)$$

where the temperature *T*(*r*,*t*) is the temperature increase as a function of the coordinate *r* and the time *t*, *k*(*r*,*t*) is the thermal conductivity (Wm^−1^∙K^−1^), *ρ* is the density(kg∙m^−3^), *C_p_* is the heat capacity (J∙kg^−1^∙K^−1^). To reach the target of destroying such a cancer cell with an internalized AuNS, it is enough to irradiate the cell with power laser of tens W/cm^2^ for 5–10 min [70]. The local heat intensity *Q*(*r*,*t*), generated by the light dissipation in AuNSs, can be expressed at the surface by

(9) Q(r,t)=ω8π|E(r=R)|2⋅Imε(r,ω)

where *R* is the AuNS radius, and *ε*(*r*,*ω*) is the AuNS dielectric function. The thermal heat and its propagation can be defined by two figures of merit [71]: the first one defined as the localization length of temperature; the second one defined as the ability of a plasmonic structure to create large temperature gradients. The first figure of merit can be quantified by the ratio ∆*L_heating_*/*L_heater_*, where *L_heater_* is the size of a plasmonic heater, i.e., NP or NS, etc. The length ∆*L_heating_* is the dimension of the heated area where the local temperature increase is above the threshold value ∆*T*_max_/2. The second figure of merit can be defined as |*dT*(*r*)/*dl*|_max_/*I*_flux_, where |d*T*(*r*)/d*l*|_max_ denotes the maximum temperature gradient and *I*_flux_ is the light flux. These two figures of merit are independent of the incident light power. In terms of absorption efficiencies, the total absorbed energy can be calculated considering *Q_abs_*∙∆*t_pulse_*, where the total illumination power density is defined as *P_tot_* = *I_flux_*∙∆*t_pulse_*, so that the efficiency between the temperature of the system and the correspondent absorption induced by the NPs is defined as ∆*T*_max_/*Q_abs_*.

## 4. Detection and Tracking Techniques of Intracellular AuNPs

There are several approaches to the detection of individual metal NPS. They are based on far-field or near-field optical properties. For example, they can exploit the generation of new wavelength by the particle under investigation, either in a linear photoluminescence- or in nonlinear-processes. Alternatively, detection can be done at the same illuminating wavelength or detecting directly the scattered light or using the interference of the scattered wave with a reference wave. In this last case, the advantage of the interference signal is that the scattered intensity varies with the sixties power. Improving the sensitivity of both absorption-based and scattering-based methods making possible also to give access to both the amplitude and the phase of the scattered wave. Such methods must be modified and improved for the detection of internalized AuNPs, as well as any other metal types of NPs, inside cells. Detection and tracking of their intracellular activity require the combination complementary combination of different techniques. Such experimental framework should be able to include quantitative assessment of uptake, NP intracellular localization, biochemical environment and the role of NP. Some of such techniques have been briefly presented in the Introduction. In this section, we wish to focus the attention specifically on the three techniques, DFM, SERS and aperture SNOM.

**Figure 2:**
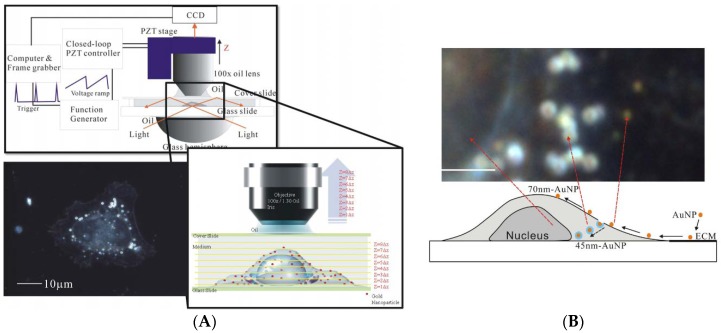
(**A**) The optical setup of the DFM microscope used by Wang et al. A light source of 60 W metal halide light illuminates the samples through a hemisphere glass lens. The scattered light is collected by an objective lens and imaged by a color CCD. The scattering images can be collected at different focal positions; (**B**) A dark-field CCD image for 45- and 70-nm-AuNPs and a CL10 cell with a focus plane fixed at the glass substrate. The AuNPs uptaken by the cells became orange with blue surroundings. (Reproduced from [76] with permission).

DFM in several different configurations has been largely used to detect AuNPs inside cells. DFM denotes microscopy methods (both in optical and electron microscopy), where unscattered beam from an image in excluded. This produces a dark background with bright objects on it. As a result, this technique is able to enhance the contrast in unstained samples. Reflection-mode DFM imaging allowed Curry et al. to study the effects on epidermal growth factor receptor (EGFR) of 60-nm AuNPs conjugated to anti EGFR [72]. Making use of analogous reflection-mode DFM, Kumar et al. were able to follow 20-nm AuNPs functionalized with an anti-actin antibody for labelling actin in live cells [73], while single 80-nm AuNP imaging and tracking have been reported by Louit et al. [74]. Patskovsky et al. have developed a new hyperspectral DFM imaging system using a scanned supercontinuum light source to track 3D density of polyethylene glycol (PEG) functionalized 100-nm diameter AuNPs targeting CD44^+^ cancer cells [75]. In an analogous way, Wang et al. studied endocytosis of AuNPs sizes (45 nm, 70 nm and 110 nm) in various cells (the human cancer cell lines, CL1-0 and HeLa) [76]. In this study, the DFM exploits the spectroscopic difference between the AuNPs and cell organelles, and a color CCD with a post-processing tool can identify the positions of AuNPs, Figure 2. They demonstrated the localization of the AuNPs, if into the cytoplasm or adhering to the membrane, the size dependence of uptake processes (optimal uptake mechanisms for 45 nm). In turn, since the most AuNPs remain bound to the cell membrane, they can be used to reconstruct the 3D cellular morphology.

**Figure 3:**
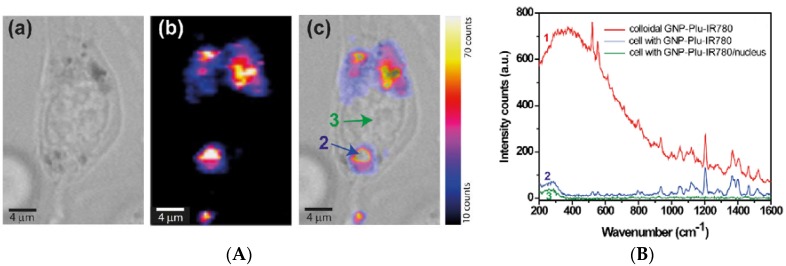
(**A**) bright field image of a C-26 cell incubated with AuNP-Plu-IR780 (a), correspondent SERS map (b) and the overlapping (c) of the two previous images; (**B**) SERS spectra of colloidal solution of AuNP-Plu-IR780 (labelled as 1), and extracted spectra from regions marked blue arrow (2) in image (**A**(c)) and from nucleus as marked with the green arrow (3) in (**A**(c)). (Reproduced from [79] with permission).

Plasmonic properties of AuNPs are the physical base for SERS-active probe for intracellular applications. One basic advantage of SERS over other imaging techniques such as fluorescence, UV-vis-NIR or Nuclear Magnetic Resonance, is the enhancement by several orders of extremely weak Raman scattering signal in proximity of AuNPs [77]. The SERS spectrum provides a complete structural characterization of target molecule functionalizing AuNPs being SERS a vibrational fingerprint, which characterizes the molecular chemical bonds and symmetry without the need for staining or expressing fluorogenic proteins [78,79]. Owing to its ultrahigh sensitivity, non-destructive character, specificity and due to the possibility to perform Raman measurements under infrared excitation SERS is readily applicable in vivo with negligible background signal [80]. By using the SERS technique in combination with near-infrared absorbing molecules in resonance with the excitation laser line, one can develop promising ultrasensitive contrast agents for in vivo cancer imaging. For this purpose, Huang et al. have reported integrin-targeted 60-nm AuNPs and gold nanostars functionalized with IR792 perchlorate and IR780 perchlorate, respectively, in a femtomolar range [81]. Nagy-Simon et al. recently provided proof-of-concept resonant SERS imaging on C-26 cell incubated with AuNP-Plu-IR780 to demonstrate the in vitro applicability of the designed NPs as SERS nanotags under near infrared excitation using 785 nm laser line [79]. In Figure 3, we report the most prominent results obtained by Nagy-Simon et al., the images denoted by Figure 3A represents, respectively, a C-26 cell, the correspondent SERS image and overlap of the previous two images. Figure 3B, instead, denotes the SERS spectrum of the AuNP-Plu(ronic)-IR780. It is relevant in Figure 3B that most of the vibrational bands collected from the cell in the presence of NPs corresponds to those present in the resonant SERS spectrum of colloidal AuNP-Plu-IR780. In addition, the intracellular NPs do not present the considerable fluorescence background (red line). This is the inherent advantage of SERS hot spots generated by clustering NPs because of their intracellular aggregation. 

In addition, SERS technique will play a key role in the development of theranostic platforms. SERS is able to perform live cell imaging in combination with DFM and DIC microscopies and to try the effects in PTT applications of designed multimodal water-soluble and stable nanosystems based on bioconjugated AuNPs [82].

**Figure 4:**
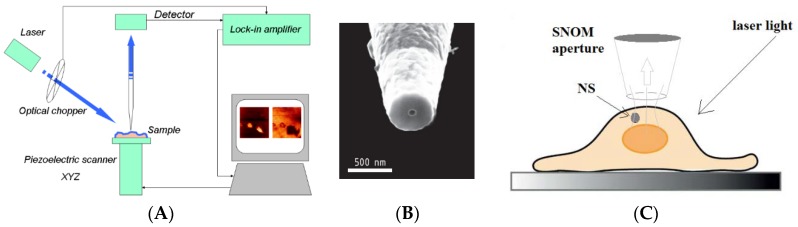
(**A**) Schematic sketch of a SNOM in collection mode; (**B**) detail of the SNOM probe; (**C**) Schematic sketch representing the physical description of dark-spot correspondent to AuNSs. The aperture SNOM is operating in collection mode, the laser light wavelength is 780 nm in order to exploit the infrared transparency window. During the scanning, when the SNOM probe is positioned in correspondence of the AuNS, the optical signal is strongly reduced. (Reprinted from [41] with permission).

However, there is another technique, which gained great advantage by plasmonic properties of AuNPs and near infrared wavelengths. This technique is the aperture SNOM. Inside the scanning probe microscopy family [83], SNOM represents the microscopy technique operating below the far-field diffraction limit by exploiting the properties of evanescent waves [84]. In a SNOM, the excitation laser light is focused through an aperture with a diameter (50–100 nm) smaller than the excitation wavelength. When the sample is scanned at a small distance (10–20 nm), the optical resolution of transmitted or reflected light is limited only by the diameters of the aperture, reaching to lateral resolution of 20 nm and vertical resolution of 2–5 nm [85]. In order to improve resolution, apertureless SNOM (aSNOM) has been designed (in some cases known as scattering SNOM) [86,87]. aSNOM is based on a dynamic mode operating AFM coupled to sophisticated interferometric optical detection system. A continuous wave laser beam is focused onto the apex of the metallic or dielectric AFM tip. The backscattered light is collected and interfered with a reference beam, which is slightly shifted with respect to the scattered beam. The interfering signals are detected by a fast avalanche photodiode. The resolution is 10 nm or better. An application of the SNOM to detect intracellular AuNSs (BaTiO3 core ~100 nm +Au shell ~40 nm) has been developed by D’Acunto et al. [38,39,40,41]. In this study, the SNOM were used in air in collection mode with an oblique angle (*θ* = 45°) illuminating laser light of 780 nm, Figure 4. This wavelength enabled the near-infrared transparency window allowing penetration depth of cm [66].

**Figure 5:**
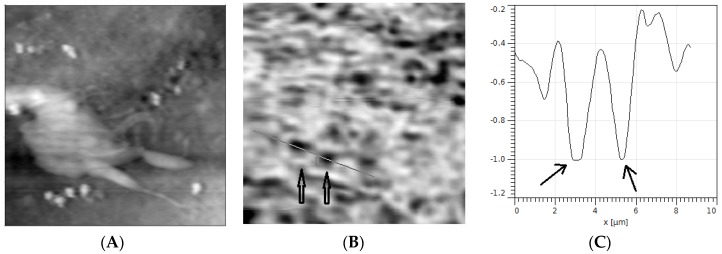
(**A**) Topography (20 μm × 20 μm × 1.5 μm) of an h9c2 mouse cell; (**B**) corresponding extinction map collected on the same area; (**C**) profile to two absorbing optical signals due to the presence of two AuNPs with an approximate transverse diameter of 150 nm, the vertical scale is in arbitrary units. The optical map was collected using a 780 nm wavelength laser light in the SNOM operating in collection mode. (Reprinted from [40] with permission).

The AuNSs employed in this experiment have an absorption efficiency greater than scattering efficiency. We therefore expect that when the instrument is measuring the cell topography, and in the same time collecting the light coming from the cell, if in the collection volume there are some AuNSs, in correspondence of which the optical map should have dark spots. Which is punctually happened, see Figure 5.

**Figure 6:**
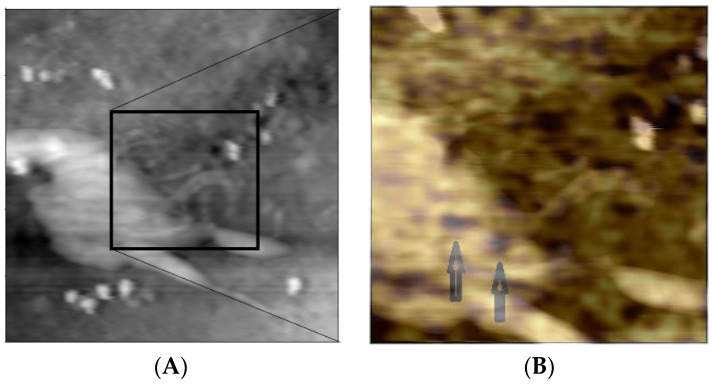
Topography (20 μm × 20 μm) (**A**) as in Figure 5 with the overlap of a zoomed area (9 μm × 9 μm) (**B**) of the optical map with the topography. The localization inside the cell of the two AuNPs is evident. (Reprinted from [41] with permission).

In order to evidence the localization of the dark spots (correspondent to AuNSs) on the topography of the cell, we overlap the topography and the correspondent optical map, Figure 6.

The volume of collection of light can be considered as a cylinder with a base area correspondent approximately to the aperture apex of the SNOM and a height of approximately less than 200 nm inside the cell [41]. This is the depth dimension of possible identification and localization of the AuNSs when an aperture SNOM is used.

Another potential method for cellular uptake and trafficking includes Super Resolution imaging [88,89,90]. Stochastic optical reconstruction microscopy (STORM) has been recently applied to the identification of polystyrene NPs [91], but its extension to AuNPs should be easy and highly recommended for improving the knowledge of cellular uptake mechanisms. STORM-based methodology, in fact, is able to resolve ~20 nm diameter NPs, probing their interactions with cellular structures by co-localization and gaining quantitative information on size, number and positioning of the internalized NPs.

AuNPs represent only one of the myriad nanomedicine tools in PTT. Other materials with different chemical and physical properties are currently employed in drug-related cancer treatments [92]. Liposomes were probably the first class of therapeutic NPs to receive clinical approval, and still today represent the basic nanoplatform in clinical-stage therapeutics. Two basic advantages of liposomes, or lipid-based NPs, are the easiness of encapsulating drug and the high tissue and cellular biocompatibility, being the cellular membrane lipid-based composition. Other materials are polymeric micelles and polymeric NPs, two new classes of multifunctional cancer therapeutic agent. All such materials have in common to AuNPs the theranostics ability. Already today and more and more in the immediate future, to all the materials used in cancer treatments new smart functionalities will be added in order to make them more effective in their interaction with both sick and healthy cells [93,94].

## 5. Concluding Remarks and Future Challenges

The use of nanoparticles in medicine is a key application requiring many multidisciplinary efforts ranging from nanotechnology to material chemistry and biophysics. Interactions between nanoparticles and cells are one of the major framework of nanomedicine. However, this key application is still challenging, and nanomedicine fails to be translated to the clinic. The crucial factor, among others, limiting the rational design of effective nanomedicine is the inadequate understanding of nanoparticle-cell interactions and consequent cellular uptake mechanisms.

Cellular uptake mechanisms play a key role in PTT. PTT is a reduced toxicity and non-invasive treatment for the therapy for many diseases, in a special manner, for local cancer treatment. It is based on the heat induced by a laser illumination able to activate the heat production by internalized NPs in cancer cells.

In this review, we have described (i) endocytosis and cellular uptake mechanisms of bared or functionalized AuNPs; (ii) the optical and photothermal properties of AuNPs describing the plasmonic properties of such NPs, in turn; (iii) some experimental techniques for detection and tracking of intracellular AuNPs.

AuNPs represent a fundamental opportunity in nanomedicine. Next challenges require both improved translational application to clinic PTT and/or super-resolution methods to best detection and tracking of intracellular AuNP probes in single cells or subcellular organs and components, eventually combined with nanospectroscopy techniques.
